# Purification, crystallization and preliminary X-ray analysis of a deletion mutant of a major buckwheat allergen

**DOI:** 10.1107/S1744309109043127

**Published:** 2009-11-27

**Authors:** Yuichiro Kezuka, Takashi Itagaki, Rie Satoh, Reiko Teshima, Takamasa Nonaka

**Affiliations:** a Department of Structural Biology, School of Pharmacy, Iwate Medical University, Yahaba, Iwate 028-3694, Japan; b Department of BioEngineering, Nagaoka University of Technology, Nagaoka, Niigata 940-2188, Japan; c Division of Novel Foods and Immunochemistry, National Institute of Health Sciences (NIHS), Setagaya, Tokyo 158-8501, Japan

**Keywords:** buckwheat allergen, 2S albumin, BWp16

## Abstract

A 16 kDa buckwheat protein (BWp16) is a major allergen responsible for immediate hypersensitivity reactions including anaphylaxis. A deletion mutant of BWp16 (rBWp16ΔN) was overproduced and purified and was shown to be immunologically active. A three-wavelength MAD data set was collected from a crystal of selenomethionine-labelled rBWp16ΔN. The crystal belonged to the triclinic space group *P*1, with unit-cell parameters *a* = 28.39, *b* = 31.54, *c* = 32.20 Å, α = 111.92, β = 108.91, γ = 98.74°. One monomer was expected to be present in the asymmetric unit based on the calculated Matthews coefficient of 1.76 Å^3^ Da^−1^.

## Abstract 2

A 16 kDa buckwheat protein (BWp16) is a major allergen responsible for immediate hypersensitivity reactions including anaphylaxis. An immunologically active mutant of BWp16 was prepared and a three-wavelength MAD data set was collected from a crystal of selenomethionine-labelled mutant protein.

## 1. Introduction

Buckwheat (*Fagopyrum esculentum*) belongs to the Polygonaceae family. Buckwheat noodles are a popular food in East Asian countries. Buckwheat-flour products are also becoming popular in Western countries as a healthy food with a high protein content. However, some people exhibit hypersensitivity mediated by an antibody IgE specific to buckwheat proteins (Wieslander, 1996 ▶; Horesh, 1972 ▶). Ingestion of a small amount of buckwheat can cause immediate hypersensitivity reactions, termed anaphylaxis. This hypersensitivity often starts in childhood and continues throughout life, in contrast to allergies to other foods such as cow’s milk (Takahashi *et al.*, 1998 ▶).

Investigators have reported that 24, 19, 16, 14, 10 and 9 kDa buckwheat proteins can react with IgE antibodies from allergic patients (Nagata *et al.*, 2000 ▶; Park *et al.*, 2000 ▶; Tanaka *et al.*, 2002 ▶; Yoshimasu *et al.*, 2000 ▶; Matsumoto *et al.*, 2004 ▶). Most of these investigations were performed using nonheated and non-pepsin-digested buckwheat extracts. However, the foods that we consume are usually cooked and are digested by proteolytic enzymes such as pepsin in our bodies. Tanaka and coworkers demonstrated that the 16 kDa buckwheat protein BWp16, a member of the 2S albumin family (Shewry *et al.*, 1995 ▶), was resistant to pepsin and preserved its reactivity with IgE antibodies from allergic patients with immediate hypersensitivity reactions (Tanaka *et al.*, 2002 ▶). In contrast, the other candidates were digested by pepsin and the resulting fragments lost their reactivity with IgE antibodies. Thus, BWp16 is a strong candidate as the major buckwheat allergen responsible for immediate hypersensitivity reactions including anaphylaxis.

The epitopes recognized by IgE antibodies are divided into two types: a linear epitope in which the key residues arise from a linear amino-acid sequence and a structural epitope in which the key residues arise from widely different positions in the linear amino-acid sequence. As mentioned above, IgE antibodies might recognize the structural epitopes of BWp16 because of its pepsin resistance. To investigate the molecular mechanism of buckwheat allergy at an atomic level, it is important to determine the structural epitopes of the major buckwheat allergen BWp16. Therefore, we attempted the structural characterization of recombinant BWp16 (rBWp16) and its N-terminal deletion mutant (rBWp16ΔN), which appears to be immunologically equivalent to BWp16. Here, we describe the purification, crystallization and preliminary X-ray analysis of rBWp16ΔN.

## 2. Materials and methods

### 2.1. Cloning, expression and purification

rBWp16 was prepared as described previously (Koyano *et al.*, 2006 ▶) and further purified using a prepacked anion-exchange column with a bed volume of 1 ml (HiTrap Q FF, GE Healthcare). rBWp16ΔN was overproduced in *Escherichia coli* as a soluble protein with a glutathione *S*-transferase (GST) tag at its N-terminus. The gene encoding residues 13–127 was amplified using the forward primer 5′-CGTGGATCC *CTGGTTCCGCGTGGA* ***AGC***TCAAAGTGCATGCGA-3′ and the reverse primer 5′-CGGCTCGAGTTACACAAAATACCGATTTCCTCT-3′. [The underlined sequences denote introduced *Bam*HI and *Xho*I sites, respectively, and the italicized sequence denotes a thrombin recognition site. The codon AGC (shown in bold in the forward primer) for the last amino acid (Ser) of the thrombin recognition site overlapped with that for the 13th amino acid of BWp16 (Ser13).] The resulting fragment was cut and cloned into pGEX-6P-2 (GE Healthcare) vector. rBWp16ΔN consisted of residues 13–127 of BWp16 and the N-terminal vector-derived Gly residue left after removal of the GST tag by thrombin cleavage. The constructed plasmid was then transformed into *E. coli* strain Origami B (DE3) (Novagen). The cells were grown at 310 K in Luria–Bertani (LB) medium. When the optical density of the medium reached 0.4 at 600 nm, isopropyl β-d-1-thiogalactopyranoside was added to a final concentration of 0.4 m*M*. Cultivation was continued for 4 h at 298 K. The cells were resuspended in phosphate-buffered saline (PBS) and then sonicated. The soluble fraction was passed through a 0.22 µm syringe filter and then bound to 5 ml of Glutathione-Sepharose FF (GE Healthcare) in an open column. The bound protein was exhaustively washed with PBS and eluted with 50 m*M* Tris–HCl pH 8.0 containing 10 m*M* reduced γ-glutathione. The GST-fusion protein was digested by thrombin and dialyzed against PBS at 293 K. To remove the digested GST tag, the digested protein was again applied onto the above-mentioned open column. The flowthrough fraction was dialyzed against 20 m*M* Tris–HCl pH 8.5 and loaded onto MonoQ HR10/10 (GE Healthcare) equilibrated with the same buffer. The bound rBWp16ΔN was eluted with a linear gradient (20 m*M* increase per column volume) of sodium chloride (NaCl) at a flow rate of 2.0 ml min^−1^. Selenomethionine (SeMet)-labelled protein was overproduced in *E. coli* strain B834 (DE3) (Novagen). It was prepared by the same procedure as described above except for the use of LeMaster medium (LeMaster & Richards, 1985 ▶) instead of LB medium.

### 2.2. Enzyme-linked immunosorbent assay (ELISA)

After coating 96-well plates (Nunc LockWell Module Plate, Nunc) with rBWp16 or rBWp16ΔN (1.0 µg per 50 µl of 50 m*M* sodium carbonate buffer pH 9.6 in the well) and incubating overnight at 277 K, they were washed with PBS containing 0.05% Tween 20 (PBS-­T) and blocked with 0.1% casein-PBS for 1 h at room tem­perature. These plates were then washed again with PBS-T and incubated overnight at 277 K with serum samples diluted to 5% (1:20 dilution) in 0.1% casein-PBS. The wells were washed four times with PBS-T containing 1.0 *M* NaCl. They were then exposed to horseradish peroxidase (HRP) conjugated goat anti-human IgE antibodies (Nordic Immunology, 1:1000 dilution) in 0.1% casein-PBS for 1 h at room temperature. The bound antibodies were reacted with a sub­strate solution (TMB reagent, BD Biosciences). The colorimetric intensity at 450 nm was measured according to the manufacturer’s protocol.

### 2.3. Crystallization

Initial screening for crystallization was performed by the hanging-drop vapour-diffusion method at 293 K. The protein solution was adjusted to 15 mg ml^−1^ (1.1 m*M*) in 10 m*M* MES pH 6.5 containing 10 m*M* NaCl. Each hanging drop was prepared by mixing 0.7 µl each of the protein solution and the reservoir solution and was equilibrated against 0.5 ml of the latter. Commercially available kits manufactured by Emerald BioSystems and Hampton Research were used for initial crystallization screening and a total of approximately 400 conditions were tried. The identified conditions were then optimized by replacing the buffer and/or the precipitant and by changing the concentrations of the precipitant, buffer and additive.

### 2.4. Data collection and processing

Crystals were mounted in cryoloops (Hampton Research) and cryoprotected by soaking them briefly in mother liquor containing 20%(*w*/*v*) 2-methyl-2,4-pentanediol before flash-freezing in a stream of nitrogen gas at 95 K. Native and three-wavelength MAD data sets were collected on beamline BL6A at the Photon Factory using a Quantum 4R detector (Area Detector Systems Corporation). The MAD experiment wavelengths were determined from an XAFS experiment on an SeMet-labelled rBWp16ΔN crystal. The diffraction data were indexed, integrated and scaled using *MOSFLM* (Powell, 1999 ▶) and *SCALA* (Collaborative Computational Project, Number 4, 1994 ▶) as implemented in *XIA*2 (http://www.ccp4.ac.uk/xia/). An anomalous difference Patterson map for the peak data set (λ = 0.9786 Å) was calculated using *FFT* (Read & Schierbeek, 1988 ▶) and contoured sections were drawn using *MAPSLICER* (Winn *et al.*, 2002 ▶).

## 3. Results and discussion

A homology search of BWp16 within the Protein Data Bank showed that BWp16 had sequence similarity to 2S albumins or related proteins from peanut (PDB code 1w2q; 30% identity; Lehmann *et al.*, 2006 ▶), castor bean (PDB code 1psy; 27% identity; Pantoja-Uceda *et al.*, 2003 ▶) and oilseed rape (PDB code 1sm7; 23% identity; Pantoja-Uceda *et al.*, 2004 ▶). These structures were determined by NMR spectroscopy; no crystal structures of 2S albumins were available. The N-terminal regions of the 2S albumins from peanut and castor bean were highly disordered. We therefore prepared an N-terminal deletion mutant BWp16ΔN as the N-terminal region of BWp16 was predicted to be flexible by analogy with the 2S albumins. rBWp16ΔN was expressed as a GST-fusion protein in *E. coli* and was purified to apparent homogeneity, yielding approximately 1.5 mg of protein per litre of medium.

To clarify whether rBWp16ΔN could react with IgE antibodies from an allergic patient, we performed ELISA. The IgE-binding activity of serum from a buckwheat-allergic patient to rBWp16ΔN was comparable with that to rBWp16. The colorimetric intensities measured at 450 nm were 0.168 ± 0.024 and 0.161 ± 0.028 for rBWp16 and rBWp16ΔN, respectively. rBWp16 was shown to be immunologically equivalent to the wild-type BWp16 (Koyano *et al.*, 2006 ▶). These results indicate that rBWp16ΔN possesses allergenicity and that the 12 N-terminal residues are not necessary for IgE binding.

**Figure 1:**
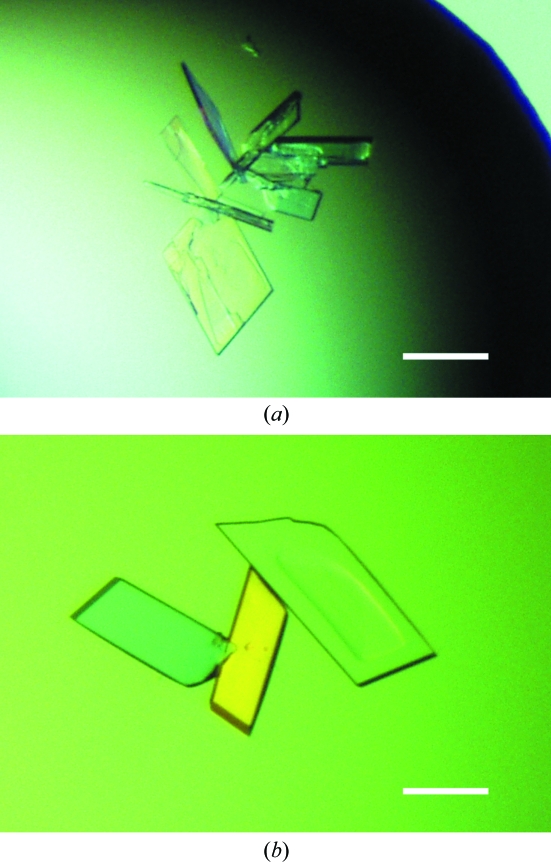
Crystals of rBWp16ΔN (*a*) and its SeMet derivative (*b*). The scale bars correspond to 0.1 mm. The crystals in (*a*) and (*b*) were obtained under conditions I and II, respectively.

After trials of approximately 400 conditions in the initial screening, an rBWp16ΔN crystal was obtained under condition No. 40 of the Cryo I kit from Emerald BioSystems [40%(*v*/*v*) ethanol, 100 m*M* phosphate–citrate pH 4.2, 5%(*w*/*v*) PEG 1000]. However, we did not succeed in crystallizing rBWp16. Therefore, the deletion of the 12 N-­terminal residues was likely to affect crystallization. Crystallization conditions were optimized for rBWp16ΔN and the following two promising conditions were established. One is a slight modification of Cryo I condition No. 40 [36%(*v*/*v*) ethanol, 0.1 *M* phosphate–citrate pH 4.2, 1%(*w*/*v*) PEG 1000; condition I]. The other was 29%(*v*/*v*) 1-­propanol, 0.1 *M* phosphate–citrate pH 4.2, 1%(*w*/*v*) PEG 1000 (condition II). Plate-like crystals grew within 2 d and reached a maximum dimension of 0.2 mm (Fig. 1 ▶ *a*); dissolution occurred within several days under both conditions. A complete data set was collected to 1.72 Å resolution from a native crystal obtained under condition I. Table 1 ▶ gives a summary of the data-collection and data-processing statistics. The native crystal belonged to the monoclinic space group *P*2_1_, with unit-cell parameters *a* = 27.92, *b* = 58.54, *c* = 32.16 Å, β = 109.34°. The calculated Matthews coefficient of 1.87 Å^3^ Da^−1^ (Matthews, 1968 ▶), corresponding to a solvent content of 34%, indicated the presence of one monomer in the asymmetric unit.

**Figure 2:**
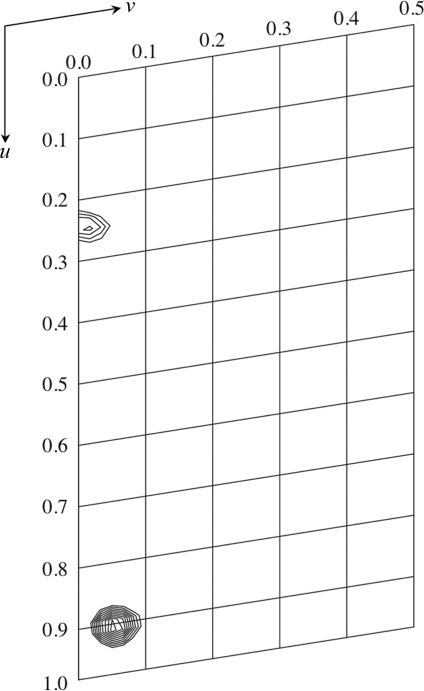
A section at *w* = 0.317 of the anomalous difference Patterson map for the SeMet derivative containing the highest peak. The map was calculated using the peak data and is contoured at intervals of 0.5σ starting at 3.0σ above the mean density level.

We first attempted to solve the structure of rBWp16ΔN using molecular-replacement (MR) techniques. Three homology models were constructed based on the above-mentioned protein structures using *MODELLER* (Sali & Blundell, 1993 ▶) and were used as search models. However, no significant peaks were found in the rotation and translation functions despite exhaustive attempts and the calculated phases from the MR solutions did not give any interpretable electron density. Therefore, we prepared SeMet-labelled rBWp16ΔN and crystallized it for MAD structure determination. The purified protein yield decreased to 0.1 mg per litre of medium for derivatization. SeMet-labelled rBWp16ΔN crystals were grown under conditions I and II. Fig. 1 ▶(*b*) shows SeMet-labelled rBWp16ΔN crystals obtained under condition II. In the XAFS experiment a clear selenium absorption edge was monitored from a crystal, enabling us to determine the peak (0.9786 Å), edge (0.9791 Å) and remote (0.9639 Å) wavelengths. A three-wavelength MAD data set was collected to 1.60 Å resolution from a crystal obtained under condition II. The crystal belonged to the triclinic space group *P*1, with unit-cell parameters *a* = 28.39, *b* = 31.54, *c* = 32.20 Å, α = 111.92, β = 108.91, γ = 98.74°. One monomer was expected to be present in the unit cell based on the calculated Matthews coefficient of 1.76 Å^3^ Da^−1^ (Matthews, 1968 ▶), corresponding to a solvent content of 30%. Table 1 ▶ shows a summary of the data-collection and data-processing statistics. The peak data set showed strong peaks derived from Se atoms in an anomalous difference Patterson map contoured at 3.0σ above the mean density level. A section of the map containing the highest peak is shown in Fig. 2 ▶. Phasing calculations using *SOLVE* (Terwilliger & Berendzen, 1999 ▶) are now in progress.

## Figures and Tables

**Table 1:** Data-collection and processing statistics Values in parentheses are for the highest resolution shell.

		SeMet derivative		
Data set	Native	Peak	Edge	Remote
Experimental conditions				
Beamline	BL6A	BL6A		
Wavelength (Å)	0.9780	0.9786	0.9791	0.9639
Temperature (K)	95	95		
Detector	ADSC Quantum 4R	ADSC Quantum 4R		
Oscillation angle (° per frame)	1.0	1.0	1.0	1.0
Exposure time (s per frame)	30.0	30.0	30.0	30.0
No. of images	180	360	300	172
Intensity statistics				
Space group	*P*2_1_	*P*1		
Unit-cell parameters (Å, °)	*a* = 27.92, *b* = 58.54, *c* = 32.16, β = 109.34	*a* = 28.39, *b* = 31.54, *c* = 32.20, α = 111.92, β = 108.91, γ = 98.74		
Software used	*MOSFLM*	*MOSFLM*		
Resolution range (Å)	30.34–1.72 (1.76–1.72)	27.81–1.60 (1.64–1.60)	27.22–1.60 (1.64–1.60)	27.21–1.60 (1.64–1.60)
*R*_merge_† (%)	4.0 (24.8)	4.0 (11.9)	3.6 (13.7)	8.6 (33.1)
Completeness (%)	99.7 (100.0)	88.0 (87.1)	88.1 (88.2)	87.1 (87.3)
*I*/σ(*I*)	12.2 (3.0)	12.8 (6.1)	14.8 (5.4)	6.8 (2.3)
Multiplicity	3.6 (3.6)	3.8 (3.8)	3.2 (3.2)	1.8 (1.9)

† *R*_merge_ = 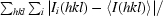 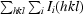, where *I_i_*(*hkl*) is the intensity of the *i*th observation and 〈*I*(*hkl*)〉 is the mean intensity of the reflections.
